# Intimate partner violence and post-traumatic stress disorder

**DOI:** 10.1192/j.eurpsy.2022.604

**Published:** 2022-09-01

**Authors:** B. Vargas, C. Santana, V. Valdéz

**Affiliations:** Universidad Católica de Santiago de Guayaquil, Ciencias De La Salud - Facultad De Medicina, Guayaquil, Ecuador

**Keywords:** mental health, latin america, Gender violence, Intimate Partner Violence

## Abstract

**Introduction:**

Post-traumatic stress disorder (PTSD), is a Mental Health condition due to a traumatic experience. There are four types of Gender Violence in Latin America: physical, sexual, psychological or patrimonial violence, when it occurs between intimate partners it is called intimate partner violence (IPV). PTSD is highly associated with IPV.

**Objectives:**

Determine the statistical index of IPV and PTSD in women and men in Guayaquil-Ecuador.

**Methods:**

We carried out a descriptive cross-sectional study, the sample was collected at the Florida Prosecutor’s Office in Guayaquil-Ecuador by UCSG medical students in 2018. The sample was 239 individuals, 195 women, 44 men. Individuals were separated into groups by gender, marital status, children, age, habits and PTSD. We applied Beck test for Depression, Davidson and DSM-5 for PTSD.

**Results:**

In this study we observe a male population suffering from IPV. Complaints of IPV 195 women (81.59%), 44 men (18.41%). PTSD positive 159 women (81.96%), men 35 (18.04%). More prevalent in age ranges 25-34. PTSD with children 147 (76%) and without children 47 (24%).

**Conclusions:**

Factors such as being a woman, having children and younger ages are linked in this study as predisposing to suffer from IPV and PTSD. We highlight a male population that reports suffering from IPV despite the lack of support, especially in Latin America. It is worth mentioning that the final consequence in many cases is femicide and homicide. Although IPV is serious and frequent, Medical professionals still do not focus on IPV diagnosis, therefore affected individuals don t receive support.

**Disclosure:**

No significant relationships.

